# Health facility readiness and provider knowledge as correlates of adequate diagnosis and management of pre-eclampsia in Kinshasa, Democratic Republic of Congo

**DOI:** 10.1186/s12913-020-05795-1

**Published:** 2020-10-07

**Authors:** Dalau Mukadi Nkamba, Roland Vangu, Moyene Elongi, Laura A. Magee, Gilbert Wembodinga, Pierre Bernard, John Ditekemena, Annie Robert

**Affiliations:** 1 Kinshasa School of Public Health, Faculty of Medicine, University of Kinshasa, Kinshasa, Democratic Republic of Congo; 2 Pôle d’Épidémiologie et Biostatistique, Université catholique de Louvain (UCLouvain), Institut de Recherche Expérimentale et Clinique (IREC), Clos Chapelle-aux-champs, 30 bte B1.30.13, 1200 Brussels, Belgium; 3 Department of Gynecology and Obstetrics, University Clinics of Kinshasa, Kinshasa, Democratic Republic of Congo; 4 Department of Gynecology and Obstetrics, Provincial General Hospital of Kinshasa, Kinshasa, Democratic Republic of Congo; 5 Department of Women and Children’s Health, School of Life Course Sciences, King’s College London, London, UK; 6 Pôle de Gynécologie et Obstétrique, Université Catholique de Louvain (UCLouvain), Institut de Recherche Expérimentale et Clinique (IREC), Brussels, Belgium

**Keywords:** Pre-eclampsia, Knowledge, Healthcare providers, Facility readiness, Kinshasa

## Abstract

**Background:**

Hypertensive disorders in pregnancy are the second most common cause of maternal mortality in the Democratic Republic of Congo (DRC), accounting for 23% of maternal deaths. This study aimed to assess facility readiness, and providers’ knowledge to prevent, diagnose, and treat pre-eclampsia.

**Methods:**

A facility-based cross-sectional study was conducted in 30 primary health centres (PHCs) and 28 referral facilities (hospitals) randomly selected in Kinshasa, DRC. In each facility, all midwives and physicians involved in maternal care provision (*n* = 197) were included. Data on facility infrastructure and providers’ knowledge about pre-eclampsia were collected using facility checklists and a knowledge questionnaire. Facility readiness score was defined as the sum of 13 health commodities needed to manage pre-eclampsia. A knowledge score was defined as the sum of 24 items about the diagnosis, management, and prevention of pre-eclampsia. The score ranges from 0 to 24, with higher values reflecting a better knowledge. The Mann-Witney U test was used to compare median readiness scores by facility type and ownership; and median knowledge scores between midwives in hospitals and in PHCs, and between physicians in hospitals and in PHCs.

**Results:**

Overall, health facilities had 7 of the 13 commodities, yielding a median readiness score of 53.8%(IQR: 46.2 to 69.2%). Although all provider groups had significant knowledge gaps about pre-eclampsia, providers in hospitals demonstrated slightly more knowledge than those in PHCs. Midwives in public facilities scored higher than those in private facilities (median(IQR): 8(5 to 12) vs 7(4 to 8), *p* = 0.03). Of the 197 providers, 91.4% correctly diagnosed severe pre-eclampsia. However, 43.9 and 82.2% would administer magnesium sulfate and anti-hypertensive drugs to manage severe pre-eclampsia, respectively. Merely 14.2 and 7.1% of providers were aware of prophylactic use of aspirin and calcium to prevent pre-eclampsia, respectively.

**Conclusion:**

Our study showed poor availability of supplies to diagnose, prevent and treat pre-eclampsia in Kinshasa. While providers demonstrated good knowledge regarding the diagnosis of pre-eclampsia, they have poor knowledge regarding its prevention and management. The study highlights the need for strengthening knowledge of providers toward the prevention and management of pre-eclampsia, and enhancing the availability of supplies needed to address this disease.

## Background

Hypertensive disorders in pregnancy (HDP), including pre-eclampsia and eclampsia (PE/E), are the second most common cause of maternal mortality worldwide, accounting for 10–15% of direct maternal deaths [1, 2]. Globally, it is estimated that 76,000 women and 500,000 newborn die from HDP annually [3]. Most of the deaths (99%) occur in low- and middle-income countries (LMICs) [4, 5]. These deaths are preventable by early detection, adequate treatment and timely delivery [6–9]. Effective interventions to address HDP exist, however, their implementation in LMICs remains challenging [10–12]. Systematic reviews and trials have shown the effectiveness of aspirin in the prevention of pre-eclampsia [10, 13–15], and the effectiveness of magnesium sulfate in the management of severe pre-eclampsia and eclampsia [11, 16, 17]. The World Health Organization (WHO) has considered magnesium sulfate as the first-line drug for eclampsia prevention and treatment for more than a decade [18]. In a review of interventions to decrease HDP-related maternal deaths, it was estimated that a package of interventions including the detection of proteinuria and hypertension in pregnancy, timely treatment of severe pre-eclampsia and eclampsia with magnesium sulfate, and early delivery would reduce HDP mortality by at least 84% in LMICs [19]. Historical data from Sri Lanka indicated that this LMIC was able to reduce the HDP-related maternal deaths from 50 to 10 deaths per 100, 000 births between 1930 and 1990s [19].

In most LMICs however, a huge number of women continue to suffer from HDP without receiving proven lifesaving interventions [6, 20–23]. Poor knowledge about pre-eclampsia among healthcare providers, in addition to the lack of adequate supplies, are contributing factors to the burden of HDP in LMICs [24–28].

According to the WHO, the maternal mortality ratio in the Democratic Republic of Congo (DRC) is estimated at 473 per 100,000 live births [29]. This is higher than one might expect based on a high rate of skilled attendance at delivery (80%), and a high rate of antenatal care attendance (89%) [30]. In 2018, HDP resulted in 841 (23%) out of the 3656 maternal deaths reported from the DRC’s maternal death surveillance system [31]. Preventing and managing HDP and HDP-related complications could contribute to lower maternal mortality in DRC, and meeting the United Nations Sustainable Development Goal 3 [32].

In a previous study in Kinshasa, Nkamba et al. [33] reported a low proportion of women screened for HDP within antenatal clinics (26.7%). The authors hypothesized that such low provision of screening for HDP could be a result of a number of factors including knowledge gaps about HDP among healthcare providers. It is essential that providers have the skills and knowledge, supplies and equipment needed to diagnose and promptly address HDP and HDP-related complications in women attending health facilities [33]. At the time of this study, there are no reliable data on the healthcare providers’ knowledge about HDP, including pre-eclampsia in Kinshasa. The purpose of this study was to assess the facility readiness in terms of availability of supplies and equipment to deal with pre-eclampsia; and the providers’ knowledge about the prevention, diagnosis, and management of pre-eclampsia.

## Methods

### Study setting

The study was conducted in Kinshasa, the capital city of the DRC. Kinshasa is divided into 35 health districts, each with an average of 12 health areas and a district hospital. A typical health area provides care for a population of 10,000–15,000 inhabitants, with an average of 6 primary health centres (PHCs) that provide basic curative and preventive services including routine antenatal care. According to the DRC’s health policy, a PHC is staffed by 2 to 4 midwives, and there should not be a physician at this level [34].

A district hospital (or Referral Health Centre where no district hospital exists) is a referral facility for PHCs and provides comprehensive emergency obstetric and neonatal care.

A provincial referral hospital and a teaching hospital that are tertiary-level facilities are also located in Kinshasa. Some PHCs refer directly to these tertiary-level facilities [34].

In DRC, PHCs are expected to manage mild pre-eclampsia using antihypertensive drugs. However, in the case of severe pre-eclampsia, PHCs should administer the initial loading dose of magnesium sulfate, then immediately transfer the women to district hospitals. The management of severe pre-eclampsia at the district hospital level includes the provision of antihypertensive drugs, and the Prichard regimen of magnesium sulfate (loading doses of 4 g IV and 10 g IM, and maintenance dosing of 5 g IM every 4 h) [35].

### Study design, population and sampling

This was a facility-based cross-sectional study. All of Kinshasa’s health facilities that provide emergency obstetric and neonatal care (EmONC) were stratified into (i) primary facilities comprising PHCs; (ii) secondary facilities comprising district hospitals and referral health centres; and (iii) tertiary facilities including the provincial referral hospital and the teaching hospital.

In each stratum, the sample size of health facilities was obtained by using the formula for a proportion within a finite population size [36] of 837 primary, 138 secondary and 2 tertiary facilities according to the data from the Kinshasa Provincial Ministry of Health.

To estimate the sample size of health facilities in each stratum, we considered a prior estimate of 23% for health facilities that have dipsticks for proteinuria testing in Kinshasa, as per the Services Availability and Readiness Assessment study (SARA) survey in DRC [37]; a margin error of 15% as per WHO recommendations [36]. We thus determined that 30 primary, 26 secondary and 2 tertiary health facilities were required, leading to a total of 58 facilities.

Both tertiary health facilities were included in the survey. However, the 30 primary and the 26 secondary facilities were selected randomly among the sampling frame of 837 and 138 facilities, respectively.

Within each selected health facility, all midwives and physicians involved in antenatal or maternity care provision, and posted during the day of the survey were included in the study (*n* = 197).

### Data collection

Twelve physicians were recruited as interviewers based on their previous experience in data collection, and two health officers as supervisors. Interviewers and supervisors were trained for five days before data collection. The training focused on the aim of the study, study procedures, and data collection techniques. A checklist for facility inventory was used to capture the availability of key supplies, drugs, and equipment needed to prevent, diagnose, and manage pre-eclampsia based on the DRC’s national guidelines. The inventory was completed as the team observed the pharmacy, laboratory, ANC, labor, and delivery areas. Each of the following health commodities was scored 1 point when available in the facility on the day of the survey, and 0 otherwise: Aspirin tablet, calcium gluconate, any fluids (saline, ringer lactate or dextrose), urine test for protein, potocols for management of PE/E, indwelling urinary catheter, blood pressure machine, stethoscope, fetoscope, oxygen cylinder and adult mask, and ambulance. One point was given for either antihypertensive drug available (nifedipine, hydralazine or methyldopa). Another point was given for either anticonvulsant available (magnesium sulfate or diazepam). We calculated the readiness score as a cumulative number of health commodities available in the health facility on the day of the survey, ranging from 0 to 13. We reported the score as a percentage of commodities available out of the 13 possible. The score ranged from 0 to 100%, with a high value reflecting better availability. We grouped facilities into three categories according to tertiles of the readiness score: low-tertile if the score ranged from 30.8 to 53.8%, middle-tertile if the score ranged from 53.9 to 69.2%, and high-tertile if the score ranged from 69.3 to 92.3%.

A knowledge questionnaire was used to assess provider knowledge about pre-eclampsia. The knowledge questionnaire was based on the WHO and DRC’s guidelines [18, 35], and previous studies which assessed providers’ knowledge and clinical judgment regarding the diagnosis and management of pre-eclampsia [23, 38, 39]. The questionnaire consisted of a written case scenario and related “yes/no” items. Each item was scored 1 if the correct answer was given by the healthcare provider, or 0 otherwise. The scenario involved a 20-year-old woman at 37 weeks of pregnancy, complaining of severe headache and blurred vision. Providers learned that the woman had a blood pressure of 160/110 mmHg and 3+ urine protein. At each step in the scenario, providers were asked how the case should be managed.

Based on DRC’s and WHO guidelines [18, 35], we calculated a knowledge score about diagnosis and management of severe pre-eclampsia by summing responses to the 12 following items: measuring blood pressure, monitoring fetal heart, proteinuria testing, naming severe pre-eclampsia as diagnosis, administering loading dose of magnesium sulfate, administering maintenance doses of magnesium sulfate, administering anti-hypertensive drugs, referring to a higher-level health facility, indwelling urinary catheter, inducing labor, monitoring magnesium sulfate toxicity, and continuing magnesium sulfate for 24 h after delivery. The score ranged from 0 to 12, with a high value reflecting better knowledge.

The questionnaire also included questions to assess providers’ knowledge regarding preventative interventions and risk factors for pre-eclampsia. The risk factors included primiparity, obesity, multiple pregnancy, maternal age, pre-eclampsia in a previous pregnancy (if parous), autoimmune disease, family history of pre-eclampsia, history of diabetes, history of chronic hypertension, and a history of renal disease [40]. The prophylactic interventions of pre-eclampsia included the use of low dose aspirin and calcium. A knowledge score about prevention of pre-eclampsia was built by summing the number of risk factors and prophylactic interventions spontaneously mentioned by the health provider. The score ranges from 0 to 12, with a high value reflecting better knowledge.

The knowledge score about diagnosis, management, and prevention of pre-eclampsia was obtained by summing the knowledge score about diagnosis and management of severe pre-eclampsia, and the knowledge score about prevention of pre-eclampsia. The score ranges from 0 to 24, with higher values reflecting a better knowledge.

The questionnaire also included data about providers’ characteristics: type (midwives or physicians), training and experience with ANC and pre-eclampsia management; and facilities’ characteristics such as ownership (private or public), location (urban or rural), and type (PHCs or hospital).

Prior to the actual data collection, the questionnaire was pilot-tested on 20 healthcare providers in 5 health facilities not participating in this study.

The questionnaire was administrated as face-to-face interviews. The data were collected between October 2017 and January 2018.

### Data analysis

Data were entered into EpiData software version 3.1 and subsequently exported into Stata version 14.1. Secondary and tertiary health facilities were grouped into one category named “hospitals”, as they all represent referral units for PHCs. There were no missing data.

We estimated the proportions of facilities having a given supply, drug or equipment by type of facilities (PHCs or hospitals), and by ownership (private or public). For each type of health provider, we estimated the proportion of those who responded correctly to a given knowledge item, by type of health facility. Proportions were compared using Pearson’s chi-square test or using Fisher’s exact test in cases where expected cell counts were below 5.

Continuous variables were described as mean and standard deviation (±SD) or as median and interquartile range (IQR). The Mann-Whitney U test was used to compare the median readiness scores between hospital and PHCs, and between private and public facilities. The test was also used to compare the median knowledge scores between midwives in hospitals and those in PHCs, between physicians in hospitals and those in PHCs, and between providers in private and in public facilities. A *P* value less than 0.05 was considered to indicate statistical significance.

## Results

Table 1 shows the characteristics of the antenatal clinics and healthcare providers included in the study, by facility type and ownership. Of the 58 health facilities, 41 (70.7%) were private and 17 (29.3%) were public. There were 30 (51.7%) primary facilities and 28 (48.3%) hospitals. The median number of first antenatal visits per month in study facilities was 50 (IQR: 37 to 63). There was a median of 8 (IQR: 5 to 16; range: 1 to 73) midwives per facility, and 2(IQR: 1 to 4; range: 0 to 48) physicians. There were more physicians in hospitals than in PHCs (median (IQR): 3 (2 to 14) vs 1 (0 to 2)), and more in public than in private facilities (median (IQR): 6 (3 to 22) vs 1 (1 to 2)). Overall 12.1% of facilities, (7.3% of private and 23.4% of public facilities), received drugs, supplies and equipment to manage pre-eclampsia from government or non-governmental organizations (NGOs).

**Table 1:** Characteristics of the 58 antenatal clinics sampled in Kinshasa, and of the 197 healthcare providers interviewed within these clinics

Characteristics	Facility Type		Facility ownership		Total (***n*** = 58)
	Hospitals (***n*** = 28)	PHCs (***n*** = 30)	Public (***n*** = 17)	Private (***n*** = 41)	
**Health facilities**					
Number of ANC pregnant women—no./mth					
Median (IQR)	56 (42–95)	42 (32–58)	43 (36–54)	53 (37–78)	50 (37–63)
Range	29–408	27–449	29–158	27–449	27–449
Number of physicians—no.					
Median (IQR)	3 (2–14)	1 (0–2)	6 (3–22)	1 (1–2)	2 (1–4)
Range	1–48	0–6	2–48	0–12	0–48
Number of midwives—no.					
Median (IQR)	10 (7–23)	7 (4–9)	22 (10–39)	7 (5–9)	8 (5–16)
Range	1–73	1–26	4–73	1–20	1–73
Receives supplies from partners—no.(%)^a^	5 (17.9)	2 (6.7)	4 (23.4)	3 (7.3)	7 (12.1)
**Healthcare providers**	**(*n*** **= 107)**	**(*n*** **= 90)**	**(*n*** **= 129)**	**(*n*** **= 68)**	**(*n*** **= 197)**
Type—no.(%)					
Midwives	56 (52.3)	66 (73.3)	26 (38.2)	96 (74.4)	122 (61.9)
Physicians	51 (47.7)	24 (26.7)	42 (61.8)	33 (25.6)	75 (38.1)
Gender—no.(%)					
Female	72 (67.3)	59 (65.6)	88 (68.2)	43 (63.2)	131 (66.5)
Male	35 (32.7)	31 (34.4)	25 (36.8)	41 (31.8)	66 (33.5)
Age—yrs					
Median (IQR)	40 (33–48)	41 (36–47)	39 (34–46)	42 (35–49)	40 (34–48)
Range	27–55	30–55	27–55	27–55	27–55
Experience within maternal health care—yrs					
Median (IQR)	7 (2–12)	7 (3–11)	6 (2–10)	8 (3–13)	7 (3–12)
Range	1–20	1–19	1–20	1–19	1–20
In-service training—no.(%)^b^	25 (23.4)	24 (26.7)	21 (30.9)	28 (21.7)	49 (24.9)

*PHCs* Primary health centres, *IQR* Interquartile range, *Mth* Month

^a^Whether facilities received drug, supplies and equipment for diagnosis, prevention and management of pre-eclampsia from the government or non-governmental organizations

^b^Whether healthcare providers declared they received in-service training regarding the management of hypertensive disorders in pregnancy

Of the 197 providers included in the study, 122 (61.9%) were midwives and 75 (38.1%) were physicians; 131 (66.5%) were female. In public facilities physicians were the most interviewed (61.8%), while in private facilities midwives were the most interviewed (74.4%). In public facilities 23 (88.5%) midwives were included in hospitals, whereas in private facilities 63 (65.6%) midwives were from PHCs (data not shown). A quarter of providers (24.9%) declared they received in-service training regarding the management of HDP. Half of the providers have been working in maternal health for more than 7 years (median = 7; IQR: 3 to 12 years; range: 1 to 20 years).

### Facility readiness

Table 2 shows the availability of each individual health commodity, and the readiness score. On the day of the survey, only half of the health facilities had protocols for the management of pre-eclampsia. Magnesium sulfate and calcium gluconate were available in 36.2% (95% CI, 24.6–49.6%) and 31.0% (95% CI, 20.2–44.4%) of facilities, respectively. There was no significant difference in the availability of magnesium sulfate between private and public facilities (35.3% vs 36.6%, *p* = 0.93), neither between hospitals and PHCs (42.9% vs 30.0%, *p* = 0.31). Calcium gluconate was significantly more available in public than in private facilities (52.9% vs 21.9%, *p* = 0.02). Less than one-third of facilities (29.3%; 95% CI: 18.8–42.6%) had aspirin. The availability of aspirin was significantly higher in public than in private facilities (47.1% vs 21.9%, *p* = 0.04), however, there was no significant difference between hospitals and PHCs (35.7% vs 23.3%, *p* = 0.3). Anti-hypertensive drugs such as methyldopa, nifedipine and hydralazine were available only in 18.9% (95% CI, 10.6–31.5%), 15.5% (95% CI, 8.1–27.6%) and 6.9% (95% CI, 2.5–17.4%) of health facilities, respectively. There were no significant differences in the availability of anti-hypertensive drugs between public and private facilities, neither between PHCs and hospitals.

**Table 2:** Availability of supplies, drugs and equipment to prevent and manage pre-eclampsia and eclampsia, according to facility type and ownership

Variable	Facility ownership			Facility type			All (***n*** = 58)
	Public (***n*** = 17)	Private (***n*** = 41)	***p***-value	Hospitals (***n*** = 28)	PHCs (***n*** = 30)	***p***-value	
Percent of facilities having the following drugs and supplies—no. (%)							
Aspirin tablet	8 (47.1)	12 (21.9)	0.04	10 (35.7)	7 (23.3)	0.30	17 (29.3)
Magnesium sulfate inj	6 (35.3)	15 (36.6)	0.93	12 (42.9)	9 (30.0)	0.31	21 (36.2)
Diazepam inj	12 (70.6)	23 (56.1)	0.30	19 (67.9)	16 (53.3)	0.26	35 (60.3)
Hydralazine inj	1 (5.9)	3 (7.3)	0.67	3 (10.7)	1 (3.3)	0.28	4 (6.9)
Nifedipine tablet	4 (23.5)	5 (12.2)	0.43	5 (17.9)	4 (13.3)	0.45	9 (15.5)
Methyldopa tablet	5 (29.4)	6 (14.6)	0.17	8 (28.6)	3 (10.0)	0.07	11 (18.9)
Calcium gluconate inj	9 (52.9)	9 (21.9)	0.02	10 (35.7)	8 (26.7)	0.46	18 (31.0)
Any fluids (saline, ringer lactate, dextrose) inj	17 (100)	38 (92.7)	0.35	28 (100.0)	27 (90.0)	0.24	55 (94.8)
Urine test for protein^a^	9 (52.9)	30 (73.2)	0.14	22 (78.5)	17 (56.7)	0.17	39 (67.2)
Potocols for management of PE/E	9 (52.9)	20 (48.8)	0.77	18 (64.3)	11 (36.7)	0.04	29 (50.0)
Indwelling urinary catheter	9 (52.9)	23 (56.1)	0.83	15 (53.6)	17 (56.7)	0.81	32 (55.2)
Percent of facilities having the following equipment—no. (%)							
Blood pressure machine	17 (100)	41 (100)	> 0.99	28 (100)	30 (100)	> 0.99	58 (100)
Stethoscope	17 (100)	39 (100)	> 0.99	28 (100)	30 (100)	> 0.99	58 (100)
Fetoscope	17 (100)	39 (100)	> 0.99	28 (100)	30 (100)	> 0.99	58 (100)
Oxygen cylinder and adult mask	5 (29.4)	2 (4.9)	0.01	5 (17.9)	2 (6.7)	0.18	7 (12.1)
Ambulances	10 (58.8)	11 (26.8)	0.02	15 (53.6)	6 (20.0)	< 0.01	14 (24.1)
Readiness score—%							
Median	69.2	53.8	0.14	69.2	53.8	0.011	53.8
Interquartile range	53.8 to 84.6	46.1 to 69.2		53.8 to 76.9	38.4 to 69.2		46.2 to 69.2
Tertile of readiness score—no.(%)			0.26			0.04	
Low tertile	6 (35.3)	24 (58.5)		10 (35.7)	20 (66.7)		30 (51.7)
Middle tertile	6 (35.3)	10 (24.4)		9 (32.1)	7 (23.3)		16 (27.6)
High tertile	5 (29.4)	7 (17.1)		9 (32.1)	3 (10.0)		12 (20.7)

*PHCs* Primary health centres, *inj* Injectable, *PE/E* Pre-eclampsia and eclampsia

^a^ Dipsticks or acetic acid

Stethoscopes and blood pressure machines were available at all facilities. Slightly more than two-thirds of facilities (67.2, 95% CI, 55.2–79.3%) had urine tests for protein. Only 24.1% of facilities (53.6% of hospitals and 20% of PHCs, *p* = < 0.01) had an ambulance stationed on their grounds. Overall, health facilities had a median 7 of the 13 health commodities, yielding a median readiness score of 53.8%(IQR: 46.2 to 69.2%). The median readiness score in public and private facilities were 69.2 and 53.8%, respectively, with no significant difference. Hospitals scored significantly higher than PHCs (69.2% vs 53.8%, *p* = 0.011). Facilities receiving health commodities form government or NGOs scored better than their counterparts, although the difference was not statistically significant (median (IQR): 61.5% (48.2 to 69.2%) vs 53.8% (46.2 to 62.9%), *p* = 0.55) (data not shown). Almost 59% of private facilities and 35% of public facilities fell into the low tertile of readiness.

### Providers’ knowledge

Table 3 shows provider knowledge regarding the diagnosis and management of severe pre-eclampsia. Overall, severe pre-eclampsia was correctly diagnosed by 91.4% (95% CI, 86.5–94.6%) of providers. Among providers who correctly diagnosed severe pre-eclampsia, however, only 43.9% (95% CI, 36.5–51.5%) would administer a loading dose of magnesium sulfate. More physicians in hospitals than in PHCs would administer a loading dose of magnesium sulfate to manage severe pre-eclampsia (58.8% vs 33.3%, *p* = 0.04). Only 10.3% (95% CI, 6.3–16.5%) of providers would monitor the magnesium sulfate toxicity.

**Table 3:** Proportion of providers responding correctly to case scenario related to diagnosis and management of severe pre-eclampsia, by provider type, facility type and ownership

Correct responses to case scenario	Midwives			Physicians			All (***n*** = 197)
	PHCs (***n*** = 66)	Hospitals (***n*** = 56)	***p***-value	PHCs (***n*** = 24)	Hospitals (***n*** = 51)	***p***-value	
Initial assessment							
Check blood pressure	87.9	94.6	0.22*	83.3	98.4	0.03	91.9
Monitor fetal heart rate	100	100	> 0.99	100	100	> 0.99	100
Assess urine for protein	42.4	46.4	0.66	54.2	39.2	0.22	44.2
Diagnosis and management of severe pre-eclampsia							
Diagnose severe pre-eclampsia	81.8	91.1	0.14	100	100	0.99	91.4
Administer loading dose of MgSO_4_^a^	31.5	47.1	0.11	33.3	58.8	0.04	43.9
Immediately refer to higher facility^a^	38.9	19.6	0.03	8.3	3.9	0.33*	19.4
Administer maintenance doses of MgSO_4_^ab^	33.3	48.8	0.18	31.8	57.1	0.48	45.5
Administer anti-hypertensive drugs^a^	68.5	84.3	0.06	83.3	94.1	0.14*	82.2
Induce labor^b^	3.3	9.8	0.37*	18.2	40.8	0.06	20
Monitor MgSO_4_ toxicity	0	9.8	0.08*	9.1	18.4	0.27*	10.3
Continue MgSO_4_ for 24 h postpartum^b^	6.1	7.3	0.61*	22.7	34.7	0.41	22.9
Knowledge score about diagnosis and management of severe pre-eclampsia							
Median (IQR)	5 (4 to 6)	5.5 (4 to 7)	< 0.01	5 (4 to 7)	6 (5 to 8)	0.03	5 (4 to 7)

*PHCs* Primary health centres, *IQR* Interquartile range, *MgSO*_*4*_ Magnesium sulfate

*Fisher exact test

^a^Among providers who diagnosed severe pre-eclampsia (n_i_: 51 midwives in hospitals and 54 in PHCs; 51 physicians in hospitals and 24 in PHCs);

^b^Among providers who did not refer to a higher level facility (n_i_: 41 midwives in hospitals and 33 in PHCs; 49 physicians in hospitals and 22 in PHCs)

The median knowledge score about diagnosis and management of severe pre-eclampsia was 5 (IQR, 4 to 7). Midwives in hospitals scored better than those in PHCs (median (IQR): 5.5 (4 to 7) vs 5 (4 to 6), *p* = < 0.01). Physicians in hospitals scored better than those in PHCs (median (IQR): 6 (5 to 8) vs 5 (4 to 7), *p* = 0.03).

Table 4 shows provider knowledge of risk factors and prevention strategies of pre-eclampsia.

**Table 4:** Providers’ knowledge toward risk factors and preventive interventions of pre-eclampsia, by provider type, facility type and ownership

Percentage of providers who mentioned the following:	Midwives			Physicians			All (***n*** = 197)
	PHCs (***n*** = 66)	Hospitals (***n*** = 56)	***p***-value	PHCs (***n*** = 24)	Hospitals (***n*** = 51)	***p***-value	
Risk factors for pre-eclampsia							
Chronic hypertension	36.4	35.7	0.94	41.7	64.7	0.06	44.2
Primiparity	18.2	41.1	< 0.01	45.8	70.6	0.04	41.6
Pre-eclampsia in previous pregnancy	15.2	32.1	0.03	54.2	49.0	0.68	33.5
Family history of pre-eclampsia	12.1	30.4	0.02	29.2	54.9	0.04	30.5
Diabetes mellitus	16.7	26.8	0.17	29.1	41.2	0.32	27.4
Obesity or overweight	27.3	23.2	0.61	20.8	31.4	0.34	26.4
Multiple pregnancy	9.1	26.8	0.01	33.3	41.7	0.48	24.3
Maternal age	12.1	21.4	0.17	29.2	29.4	0.98	21.3
Chronic renal disease	4.6	5.4	0.58*	12.5	19.6	0.34*	9.6
Autoimmune disease	0	1.8	0.46*	0	5.9	0.31*	2.3
Preventive intervention of pre-eclampsia							
Low-salt diet	71.2	67.9	0.69	58.3	47.1	0.36	62.4
Physical activity	24.2	21.4	0.71	25.0	5.9	0.03*	18.8
Low-dose aspirin	3.0	8.9	0.16*	20.8	31.4	0.34	14.2
Calcium	3.1	3.6	0.87	8.3	15.7	0.38	7.1
Knowledge score about prevention of pre-eclampsia							
Median (IQR)	1 (0 to 2)	2 (1 to 4)	0.01	3 (2 to 5)	4 (2 to 6)	0.16	2 (1 to 4)

*PHCs* Primary health centres

*Fisher exact test

Overall, 44.2% of providers mentioned chronic hypertension as a risk factor for pre-eclampsia. Low-dose aspirin and calcium were cited as prophylactic drugs of pre-eclampsia by only 14.2% (95% CI, 9.7–19.9%) and 7.1% (95% CI, 3.9–11.6%) of providers, respectively. Almost two-thirds of providers (62.4%; 95% CI, 55.3–69.2%) mentioned the low-salt diet as a prophylaxis for pre-eclampsia. The median knowledge score about the prevention of pre-eclampsia was 2 (IQR, 1 to 4). Midwives in hospitals were more knowledgeable than in PHCs (median (IQR): 2 (1 to 4) vs 1 (0 to 2), *p* = 0.01).

**Fig. 1:**
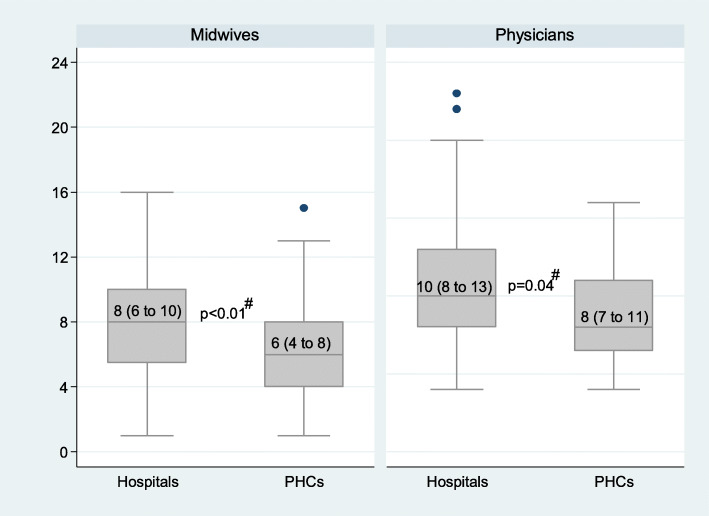
Median knowledge score about diagnosis, management and prevention of pre-eclampsia among midwives in hospitals and PHCs, and among physicians in hospitals and PHCs, within 58 antenatal clinics in Kinshasa. n_i_: 56 midwives in hospitals and 66 in PHCs; 51 physicians in hospitals and 24 in PHCs. For each box, the bottom and the top represent the first and the third quartiles, respectively. The horizontal line within the box represents the median. Numbers inside the box are median (interquartile range). The I bars represent 1.5 times interquartile range. The black dots refer to outliers. ^#^P-value from the Mann-Whitney test comparing the median knowledge scores between midwives in hospitals and those in PHCs; and between physicians in hospitals and those in PHCs

**Fig. 2:**
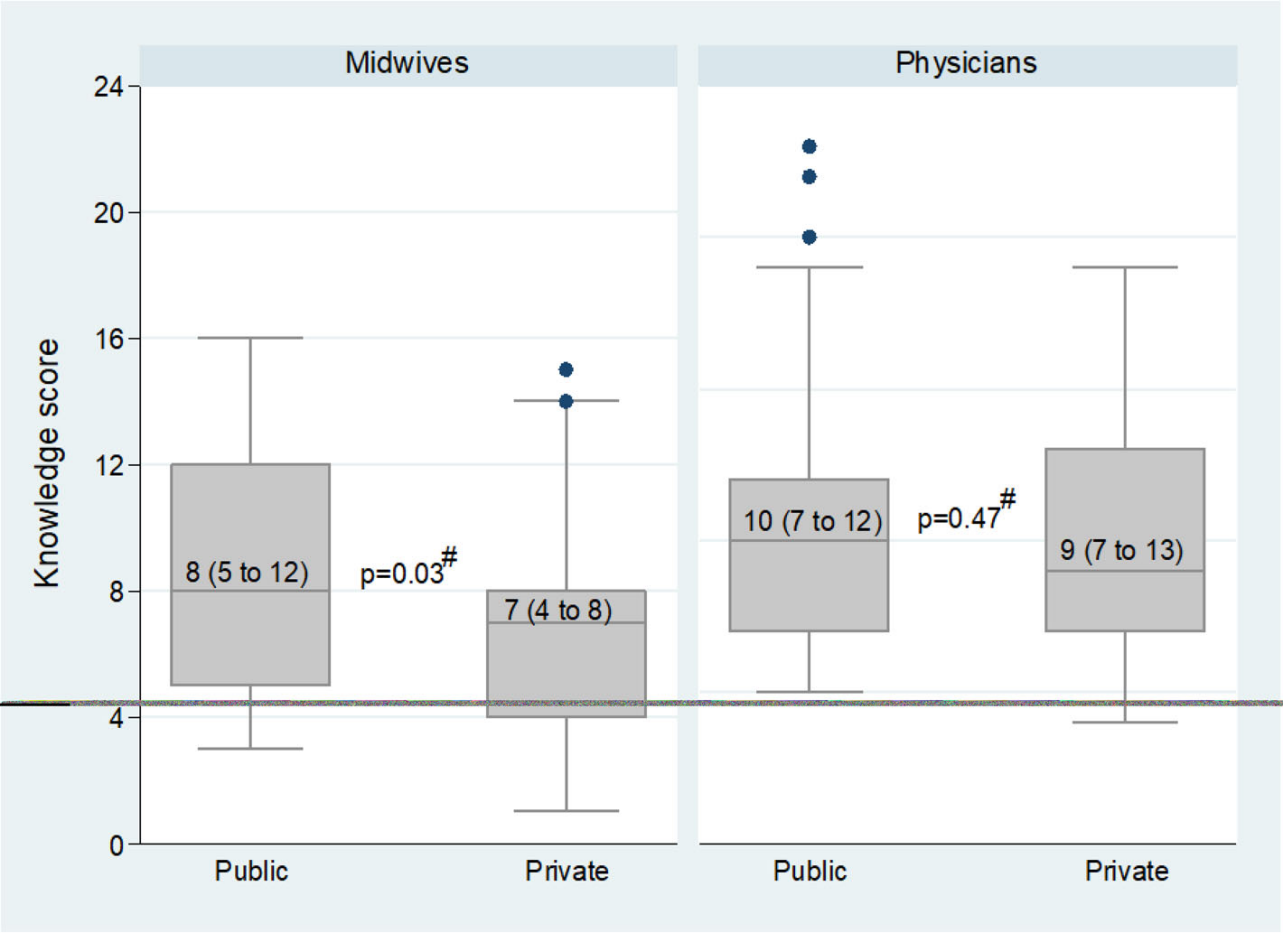
Median knowledge score about diagnosis, management and prevention of pre-eclampsia among midwives in public and private facilities, and among physicians in in public and private facilities, within 58 antenatal clinics in Kinshasa. n_i_: 26 midwives in public and 96 in private facilities; 42 physicians in apublic and 33 in private facilities. For each box, the bottom and the top represent the first and the third quartiles, respectively. The horizontal line within the box represents the median. Numbers inside the box are median (interquartile range). The I bars represent 1.5 times interquartile range. The black dots refer to outliers. ^#^*P*-value from the Mann-Whitney test comparing the median knowledge score between midwives in public and those in private facilities; and between physicians in public and those in private facilities

Figures 1 and 2 present the combined knowledge score by facility type and ownership, respectively. The knowledge score about diagnosis, management, and prevention of pre-eclampsia was higher among midwives in hospitals than in PHCs (median (IQR): 8 (6 to 10) vs 6 (4 to 8), *p* < 0.01), and higher among physicians in hospitals than in PHCs (median (IQR): 10 (8 to 13) vs 8 (7 to 11), *p* = 0.04) (Fig. 1). Midwives in public facilities were significantly more knowledgeable than in private facilities (median (IQR): 8 (5 to 12) vs 7 (4 to 8), *p* = 0.03). There was no significant difference between physicians in public and in private facilities (Fig. 2).

## Discussion

We undertook a cross-sectional study to assess readiness to diagnose, prevent and treat pre-eclampsia in health facilities in Kinshasa. This included an evaluation of critical resources for diagnosis, prevention and treatment, and provider knowledge about pre-eclampsia. Our findings showed gaps in the availability of supplies and equipment required to prevent and treat pre-eclampsia, as well as poor knowledge among healthcare providers.

Our study reveals poor availability of health commodities to manage pre-eclampsia in Kinshasa, as half of facilities had a readiness score lower than 53.8%. No facility had all the nominated commodities at the time of the study. This poor availability may be due to inconsistent supply chain, as only 12.1% of facilities, mainly hospitals (17.9%) and public facilities (23.4%), received these commodities from government or NGOs. As a result, health facilities usually ask patients or their relatives to purchase drugs from outside pharmacies [41]. As such, there may be a lag of time till the drug is made available, depending on affordability and proximity to the pharmacies. This delay may worsen the pregnancy outcome, and it contributes to HDP-related mortality [28, 42, 43]. The tendency to ask patients or their relatives to buy drugs from outside the health facilities has been also reported in other sub-Saharan African countries [28, 44].

The higher availability of health commodities in hospitals than in PHCs in Kinshasa was expected, as referral of pre-eclamptic patients to a hospital is a recommended national strategy in DRC [35]. Furthermore, the DRC’s guidelines are conflicting, with the national list of essential medicines restricting magnesium sulfate availability only in hospitals, while the EmONC guidelines recommend its availability in both PHCs and hospitals [45, 46]. Such conflicting recommendations are likely to result in poor availability of drugs in PHCs and need to be addressed.

Previous studies in Africa have reported various patterns of the available maternal health commodities. Some of these studies have favored the availability of supplies in public facilities, whereas others reported a higher availability in private facilities [47–50]. For instance, a study in Kenya, Uganda and Zambia reported a higher availability of magnesium sulfate and of calcium gluconate in public than in private facilities [48].

In our study public facilities showed better availability of commodities than private facilities, and facilities receiving health commodities from government or NGOs showed better availability than their counterparts, although differences were not statistically significant. This lack of difference in the availability of commodities should be interpreted cautiously as the study was not originally powered to capture such differences.

Despite being on the Congolese essential medicines list [46], aspirin, magnesium sulfate, calcium gluconate, and antihypertensive agents such as nifedipine, methyldopa, and hydralazine, were scarcely available in health facilities in Kinshasa. The availability of drugs such as magnesium sulfate in Kinshasa was similar to that quoted in Nigeria (34%) [44] but lower than those reported in other LMIC settings [39, 51, 52].

The availability of proteinuria testing in Kinshasa improved from 23% in 2014 to 67.2% in 2018. However, in a previous study, Nkamba et al. reported that only 26.9% of pregnant women were screened for proteinuria during a first antenatal visit [33].

The shortage of antihypertensive drugs and magnesium sulfate in health facilities, especially in PHCs, is concerning. In the DRC’s health system, PHCs serve as the first point of care facilities. They are expected to initiate antihypertensive and anticonvulsant treatment as early as possible after a diagnosis of severe pre-eclampsia, before the transfer of women to referral facilities [35]. Anti-hypertensive drugs and magnesium sulfate should be widely available at this level, so that women suffering from pre-eclampsia can be stabilized and referred safely. At both PHCs and hospital level, supplies should be available to avoid delay in treatment.

Our findings also indicate that PHCs face challenges in referring pre-eclamptic women to hospitals, as only a fifth have ambulance. It is unclear in the national guidelines if PHCs should have or should request an ambulance from hospitals when needed [34], yet the availability of ambulance at the hospital level is low. The lack of ambulance in PHCs may result in delay in women with severe pre-eclampsia being transported to referral facilities, and contribute to the HDP-related mortality.

Only one facility in two had protocols for diagnosis and management of HDP, including the administration of magnesium sulfate and anti-hypertensive drugs. Our findings are in line with those reported in other LIMCs, suggesting that the lack of protocols for the management of HDP is a common issue in these settings [6, 44, 53].

However, none of the protocols in Kinshasa included the identification of women at risk of developing pre-eclampsia during antenatal care, nor the prophylactic use of low-dose aspirin in such women.

According to the DRC’s health policy, the presence of a physician in PHCs is not mandatory [34]. Our study indicates that half of PHCs in Kinshasa, mainly private, have more than one physician. The presence of physicians in PHCs reflects the poor regulation of the DRC’s health system as reported by previous studies [54].

The proportion of providers who correctly diagnosed severe pre-eclampsia in our study is higher than that reported in India (78.5%) [38]. Although 91.4% of providers correctly diagnosed severe pre-eclampsia based on the case scenario, less than half of them identified treatment with magnesium sulfate as an essential action. The study outlined the discrepancy between the diagnosis and the management of severe pre-eclampsia, suggesting that a substantial number of women with this obstetrical complication may not receive magnesium sulfate, the drug of choice to prevent eclampsia in pre-eclamptic women, nor antihypertensive agents even when they reach health facilities in a timely manner. Our findings are consistent with studies from other settings such as India, Afghanistan, Nigeria that also reported the knowledge gaps between diagnosis and management of severe pre-eclampsia among healthcare providers [38, 39, 44, 55].

When magnesium sulfate is administered, providers should monitor women for warning signs of its toxicity, which may present as loss of tendon reflex, decreased urine output, or depressed respiratory rate [18, 40]. Our findings suggest that magnesium sulfate is not used adequately in Kinshasa, as only 10% of providers across all facilities would monitor its toxicity, and only 23% would continue its administration up to 24 h’ post-partum as per WHO and DRC’s national guidelines [18, 35].

It is known from the literature that early delivery in severe pre-eclamptic women can contribute to improving pregnancy outcomes [8, 19]. While the DRC’s national guidelines recommend inducing labor at 37 weeks gestation in case of severe pre-eclampsia, the majority of providers in Kinshasa did not identify inducing labor as a critical step in managing severe pre-eclampsia.

Although all provider groups had significant knowledge gaps about pre-eclampsia, providers in hospitals demonstrated slightly more knowledge compared to those in PHCs. This finding is not surprising given that physicians are more plentiful in hospitals than in PHCs and they tend to be more knowledgeable than midwives. National EmONC guidelines are more available in hospitals than in PHCs. Moreover, referral of pre-eclamptic patients to a hospital is a recommended national strategy in DRC. Providers in hospital may have more exposure to patients with pre-eclampsia, and thus more experience than those in PHCs.

A study from Nigeria also identified knowledge gaps about pre-eclampsia across providers both in PHCs and in hospitals [44].

Midwives in public facilities were more knowledgeable than those in private facilities. One possible explanation is that in public facilities 88.5% of midwives included in the study were from hospitals, whereas in private facilities midwives were mostly from PHCs. In addition, more providers in public facilities than in private facilities received in-service training regarding the management of HDP. There was no significant difference in knowledge between physicians in public and in private facilities. This lack of difference between physicians in public and those in private facilities may result from public-on-private dual practice among physicians, a common practice in Kinshasa, whereby some physicians from public sector are also involved in private facilities to supplement low government salary rates.

In LMICs where biomarkers to identify women at risk of developing pre-eclampsia are not available, scientific bodies recommend relying upon maternal historical and clinical characteristics [40]. The International Federation of Gynecology and Obstetrics (FIGO) has recommended a combination of i) maternal risk factors with ii) mean arterial pressure where it is not possible to measure placental growth factor (PLGF) and uterine artery pulsatility index (UTPI) [56]. In DRC however, there is a lack of consistent national guidelines regarding the prevention of pre-eclampsia, including the identification of women at risk of developing the disease, and the prophylactic use of low-dose aspirin [45]. As a result, slightly more than half (56%) of providers in our study were not aware of maternal risk factors for pre-eclampsia. Only 14.2 and 7.1% of providers named low-dose aspirin and calcium as prophylactic drugs for pre-eclampsia, respectively. Our findings regarding the prophylactic use of aspirin are similar to those reported in Nigeria (14%) but higher than those reported in Bangladesh (2%). Expectedly, the majority of providers (62.4%) mentioned the low-salt diet as a preventative strategy of pre-eclampsia. This is probably because salt restriction is a valid recommendation for hypertensive patients in general. However, scientific bodies including the WHO do not recommend salt restriction during pregnancy to prevent pre-eclampsia [57, 58].

Our findings suggest that even when women attend antenatal clinics, they may not be identified as at risk of developing pre-eclampsia. Also, even if identified as being at risk, they may not receive a preventative intervention such as low-dose aspirin, resulting in missed opportunities to prevent pre-eclampsia.

In a city such as Kinshasa, most women may choose or are forced to attend private facilities as the latter represent more than 60% of all facilities [49]. Given the knowledge gaps and shortages of supplies in both public and private facilities, it is not surprising that pre-eclampsia-related morbidity and mortality are a significant public health problem in Kinshasa. Our study highlighted the need for strengthening providers’ knowledge about pre-eclampsia, with a particular emphasis on its prevention and management. The study also highlighted the need for addressing contradictions in the DRC’s national guidelines regarding the place of availability of essential drugs and enhancing the availability of supplies and their utilization. In a recently published cluster randomized controlled trial in Kinshasa and Lusaka (Zambia), Althabe et al. showed that a combination of provision of supplies with a behavioral intervention including supportive supervision, the use of opinion leaders, reminders, audit, and feedback, improved the diagnosis and management of maternal conditions such as syphilis [59]. A similar approach could be implemented to improve the quality of care for pre-eclampsia in Kinshasa. Providers’ knowledge should be improved by means of evidence-based capacity-building strategies such as simulation-based training that have been proven to be effective in other LMICs [60, 61].

Our study has some limitations. We used a case scenario to assess providers’ knowledge to prevent and manage pre-eclampsia. Direct observation of clinical practices and case sheet audits would provide a more accurate assessment of provider knowledge. However, this strategy was not feasible in the context of DRC, where clinical records are generally of poor quality [62], and facilities, particularly PHCs do not experience a high volume of cases.

Despite these limitations, this is to our knowledge, the first study that assesses providers’ knowledge regarding the prevention and management of pre-eclampsia within a large and representative sample in Kinshasa. While this study was conducted only in Kinshasa, we feel it may also reflect the reality of health facilities in other provinces of the DRC. We encourage similar studies in other provinces of DRC for confirmation. The results of our study may be used to build strategies to improve the quality of care for pre-eclamspia in Kinshasa.

## Conclusion

Our study showed poor availability of supplies and equipment to diagnose, prevent and treat pre-eclampsia within health facilities in Kinshasa. While healthcare providers demonstrated good knowledge regarding the diagnosis of pre-eclampsia, they have poor knowledge regarding its prevention and management. The study highlighted the need for strengthening knowledge of providers toward the prevention and management of pre-eclampsia, and enhancing the availability of equipment, and supplies needed to diagnose and address this life-threatening condition.

## Data Availability

The datasets used during the current study are available from the corresponding author on reasonable request.

## Abbreviations

ANC: Antenatal care
CI: Confidence interval
DHS: Demographic and Health Survey
DRC: Democratic Republic of Congo
EmONC: Emergency obstetric and neonatal care
HDP: Hypertensive disorder in pregnancy
IQR: Interquartile range
LMIC: Low- and Middle-Income Country
MgSO_4_: Magnesium sulfate
NHIS: National Health Information System
NICE: National Institute for Health and Clinical Excellence
PHCs: Primary health centres
SARA: Service Availability and Readiness Assessment study
SD: Standard deviation

## References

[1] Kinney MV, Kerber KJ, Black RE, Cohen B, Nkrumah F, Coovadia H (2010). Sub-Saharan Africa’s Mothers, newborns, and children: where and why do they die?. PLoS Med.

[2] Say L, Chou D, Gemmill A, Tunçalp Ö, Moller A-B, Daniels J (2014). Global causes of maternal death: a WHO systematic analysis. Lancet Glob Health.

[3] Salam RA, Das JK, Ali A, Bhaumik S, Lassi ZS (2015). Diagnosis and management of preeclampsia in community settings in low and middle-income countries. J Fam Med Prim Care.

[4] Moodley J (2008). Maternal deaths due to hypertensive disorders in pregnancy. Best Pract Res Clin Obstet Gynaecol.

[5] Ghulmiyyah L, Sibai B (2012). Maternal mortality from preeclampsia/eclampsia. Semin Perinatol.

[6] Firoz T, Sanghvi H, Merialdi M, von Dadelszen P (2011). Pre-eclampsia in low and middle income countries. Best Pract Res Clin Obstet Gynaecol.

[7] Goldenberg RL, Jones B, Griffin JB, Rouse DJ, Kamath-Rayne BD, Trivedi N (2015). Reducing maternal mortality from preeclampsia and eclampsia in low-resource countries--what should work?. Acta Obstet Gynecol Scand.

[8] Goldenberg RL, McClure EM, Macguire ER, Kamath BD, Jobe AH (2011). Lessons for low-income regions following the reduction in hypertension-related maternal mortality in high-income countries. Int J Gynaecol Obstet Off Organ Int Fed Gynaecol Obstet.

[9] Moodley J (2011). Maternal deaths associated with hypertension in South Africa: lessons to learn from the Saving Mothers report, 2005–2007. Cardiovasc J Afr.

[10] Rolnik DL, Wright D, Poon LC, O’Gorman N, Syngelaki A, de Paco Matallana C (2017). Aspirin versus placebo in pregnancies at high risk for preterm preeclampsia. N Engl J Med.

[11] Altman D, Carroli G, Duley L, Farrell B, Moodley J, Neilson J (2002). Do women with pre-eclampsia, and their babies, benefit from magnesium sulphate? The Magpie Trial: a randomised placebo-controlled trial. Lancet Lond Engl.

[12] Moore GS, Allshouse AA, Post AL, Galan HL, Heyborne KD (2015). Early initiation of low-dose aspirin for reduction in preeclampsia risk in high-risk women: a secondary analysis of the MFMU High-Risk Aspirin Study. J Perinatol.

[13] Greene MF, Solomon CG (2017). Aspirin to prevent preeclampsia. N Engl J Med.

[14] Wright D, Rolnik DL, Syngelaki A, de Paco Matallana C, Machuca M, de Alvarado M (2018). Aspirin for evidence-based preeclampsia prevention trial: effect of aspirin on length of stay in the neonatal intensive care unit. Am J Obstet Gynecol.

[15] Poon LC, Rolnik DL, Tan MY, Delgado JL, Tsokaki T, Akolekar R (2018). ASPRE trial: incidence of preterm pre-eclampsia in patients fulfilling ACOG and NICE criteria according to risk by FMF algorithm. Ultrasound Obstet Gynecol Off J Int Soc Ultrasound Obstet Gynecol.

[16] Berhan Y, Berhan A (2015). Should magnesium sulfate be administered to women with mild pre-eclampsia? A systematic review of published reports on eclampsia. J Obstet Gynaecol Res.

[17] Okonofua FE, Ogu RN, Fabamwo AO, Ujah IO, Chama CM, Archibong EI (2013). Training health workers for magnesium sulfate use reduces case fatality from eclampsia: results from a multicenter trial. Acta Obstet Gynecol Scand.

[18] World Health Organization (2017). Managing complications in pregnancy and childbirth: a guide for midwives and doctors.

[19] Ronsmans C, Campbell O (2011). Quantifying the fall in mortality associated with interventions related to hypertensive diseases of pregnancy. BMC Public Health.

[20] Long Q, Oladapo O, Leathersich S, Vogel J, Carroli G, Lumbiganon P (2017). Clinical practice patterns on the use of magnesium sulphate for treatment of pre-eclampsia and eclampsia: a multi-country survey. BJOG.

[21] Lumala A, Sekweyama P, Abaasa A, Lwanga H, Byaruhanga R. Assessment of quality of care among in-patients with postpartum haemorrhage and severe pre-eclampsia at st. Francis hospital nsambya: a criteria-based audit. BMC Pregnancy Childbirth. 2017;17 https://www.ncbi.nlm.nih.gov/pmc/articles/PMC5237263/. Accessed 20 Dec 2019. 10.1186/s12884-016-1219-y PMC5237263 28086822

[22] Ali P, Butt S, Hossain N (2018). Criteria based audit in the management of eclampsia at a public sector tertiary care hospital in Karachi, Pakistan. Pregnancy Hypertens.

[23] Bazant E, Rakotovao JP, Rasolofomanana JR, Tripathi V, Gomez P, Favero R (2013). Quality of care to prevent and treat postpartum hemorrhage and pre-eclampsia/eclampsia: an observational assessment in Madagascar’s hospitals. Med Sante Trop.

[24] Sheikh S, Qureshi RN, Khowaja AR, Salam R, Vidler M, Sawchuck D (2016). Health care provider knowledge and routine management of pre-eclampsia in Pakistan. Reprod Health.

[25] Brenner S, De Allegri M, Gabrysch S, Chinkhumba J, Sarker M, Muula AS. The quality of clinical maternal and neonatal healthcare – a strategy for identifying ‘routine care signal functions’. PLoS One. 2015;10(4) https://www.ncbi.nlm.nih.gov/pmc/articles/PMC4398438/. Accessed 20 Dec 2019. 10.1371/journal.pone.0123968 PMC4398438 25875252

[26] Zuleta-Tobón JJ, Pandales-Pérez H, Sánchez S, Vélez-Álvarez GA, Velásquez-Penagos JA (2013). Errors in the treatment of hypertensive disorders of pregnancy and their impact on maternal mortality. Int J Gynaecol Obstet Off Organ Int Fed Gynaecol Obstet.

[27] Moodley J, Pattinson RC, Fawcus S, Schoon MG, Moran N, Shweni PM (2014). The confidential enquiry into maternal deaths in South Africa: a case study. BJOG Int J Obstet Gynaecol.

[28] Mahran A, Fares H, Elkhateeb R, Ibrahim M, Bahaa H, Sanad A, et al. Risk factors and outcome of patients with eclampsia at a tertiary hospital in Egypt. BMC Pregnancy Childbirth. 2017;17 https://www.ncbi.nlm.nih.gov/pmc/articles/PMC5741945/. Accessed 20 Dec 2019. 10.1186/s12884-017-1619-7 PMC5741945 29272998

[29] WHO (2019). Trends in maternal mortality 2000 to 2017: estimates by WHO, UNICEF, UNFPA, World Bank Group and the United Nations Population Division.

[30] Ministère du Plan et Suivi de la Mise en œuvre de la Révolution de la Modernité (MPSMRM), Ministère de la Santé Publique, ICF International (2014). Enquête Démographique et de Santé en République Démocratique du Congo 2013–2014.

[31] République Démocratique du Congo. Ministère de la Santé (2018). Bulletin N°1 de la surveillance des décès maternels et riposte (SDMR).

[32] United Nations General Assembly. Transforming our world: the 2030 agenda for sustainable development. New York; 2015. http://www.un.org/ga/search/view_doc.asp?symbol=A/RES/70/1&Lang=E. Accessed 20 Dec 2019.

[33] Nkamba DM, Ditekemena J, Wembodinga G, Bernard P, Tshefu A, Robert A (2019). Proportion of pregnant women screened for hypertensive disorders in pregnancy and its associated factors within antenatal clinics of Kinshasa, Democratic Republic of Congo. BMC Pregnancy Childbirth.

[34] République Démocratique du Congo. Ministère de la Santé. Recueil des normes de la zone de sante. Kinshasa; 2006. https://www.who.int/hac/techguidance/training/analysing_health_systems/5_normes_de_la_zone_de_sante_06.pdf. Accessed 20 Dec 2019.

[35] République Démocratique du Congo (2012). Normes de la zone de sante relatives aux interventions intégrées de santé de la mère, du nouveau-né et de l’enfant en République Démocratique du Congo.

[36] World Health Organization. Service Availability and Readiness Assessment (SARA). An annual monitoring system for service delivery. Implementation guide: World Health Organization; 2013. https://apps.who.int/iris/bitstream/handle/10665/112798/WHO_HIS_HSI_RME_2013_2_eng.pdf;jsessionid=A3329C22896BF6EB61E2111C7256B4F8?sequence=1. Accessed 20 Dec 2019.

[37] Ministère de la Santé Publique. Enquête sur la disponibilité et la capacité opérationnelle des services de santé en République Démocratique du Congo. Kinshasa: DSSP/DSNIS; 2014.

[38] Jayanna K, Mony P, Ramesh BM, Thomas A, Gaikwad A, Mohan HL (2014). Assessment of facility readiness and provider preparedness for dealing with postpartum haemorrhage and pre-eclampsia/eclampsia in public and private health facilities of northern Karnataka, India: a cross-sectional study. BMC Pregnancy Childbirth.

[39] Ansari N, Manalai P, Maruf F, Currie S, Stekelenburg J, van Roosmalen J (2019). Quality of care in early detection and management of pre-eclampsia/eclampsia in health facilities in Afghanistan. BMC Pregnancy Childbirth.

[40] Brown MA, Magee LA, Kenny LC, Karumanchi SA, McCarthy FP, Saito S (2018). The hypertensive disorders of pregnancy: ISSHP classification, diagnosis & management recommendations for international practice. Pregnancy Hypertens.

[41] Mondo TMN, Malengreau M, Kayembe Kalambayi P, Lapika Dimomfu B (2010). Delays in seeking and getting care, in seriously ill women of childbearing age in Kinshasa. Rev Epidemiol Sante Publique.

[42] Thaddeus S, Maine D (1994). Too far to walk: maternal mortality in context. Soc Sci Med 1982.

[43] Sk MIK, Paswan B, Anand A, Mondal NA. Praying until death: revisiting three delays model to contextualize the socio-cultural factors associated with maternal deaths in a region with high prevalence of eclampsia in India. BMC Pregnancy Childbirth. 2019;19 https://www.ncbi.nlm.nih.gov/pmc/articles/PMC6712765/. Accessed 20 Dec 2019. 10.1186/s12884-019-2458-5 PMC6712765 31455258

[44] Warren C, Ishaku S, Oginni AB, Adoyi G, Kirk KR, Dempsey A (2015). Landscaping analysis for pre-eclampsia and eclampsia in Nigeria.

[45] République Démocratique du Congo, Ministère de la Santé Publique. Normes et directives des interventions intégrées de santé de la mère, du nouveau-né et de l’enfant en République Démocratique du Congo. volume 1 soins: obstétricaux essentiels. Kinshasa; 2012.

[46] République Démocratique du Congo, Ministère de laSanté Publique (MSP). Liste nationale des médicaments essentiels. Révision Juin 2014. Kinshasa: MSP; 2016.

[47] Hanson C, Gabrysch S, Mbaruku G, Cox J, Mkumbo E, Manzi F (2017). Access to maternal health services: geographical inequalities, United Republic of Tanzania. Bull World Health Organ.

[48] Ooms GI, Kibira D, Reed T, van den Ham HA, Mantel-Teeuwisse AK, Buckland-Merrett G. Access to sexual and reproductive health commodities in East and Southern Africa: a cross-country comparison of availability, affordability and stock-outs in Kenya, Tanzania, Uganda and Zambia. BMC Public Health. 2020;20 https://www.ncbi.nlm.nih.gov/pmc/articles/PMC7333276/. Accessed 15 July 2020. 10.1186/s12889-020-09155-w PMC7333276 32620159

[49] Droti B, O’Neill KP, Mathai M, Dovlo DY, Robertson J. Poor availability of essential medicines for women and children threatens progress towards Sustainable Development Goal 3 in Africa. BMJ Glob Health. 2019;4(Suppl 9) https://www.ncbi.nlm.nih.gov/pmc/articles/PMC6797404/. Accessed 15 July 2020. 10.1136/bmjgh-2018-001306 PMC6797404 31673436

[50] Basu S, Andrews J, Kishore S, Panjabi R, Stuckler D. Comparative performance of private and public healthcare systems in low- and middle-income countries: a systematic review. PLoS Med. 2012;9(6) https://www.ncbi.nlm.nih.gov/pmc/articles/PMC3378609/. Accessed 15 July 2020. 10.1371/journal.pmed.1001244 PMC3378609 22723748

[51] Vousden N, Lawley E, Seed PT, Gidiri MF, Goudar S, Sandall J, et al. Incidence of eclampsia and related complications across 10 low- and middle-resource geographical regions: secondary analysis of a cluster randomised controlled trial. PLoS Med. 2019;16(3) https://www.ncbi.nlm.nih.gov/pmc/articles/PMC6440614/ Accessed 20 Dec 2019. 10.1371/journal.pmed.1002775 PMC6440614 30925157

[52] Katageri G, Charantimath U, Joshi A, Vidler M, Ramadurg U, Sharma S (2018). Availability and use of magnesium sulphate at health care facilities in two selected districts of North Karnataka, India. Reprod Health.

[53] Williams A, Khan MA, Moniruzzaman M, Rahaman ST, Mannan II, de Graft-Johnson J (2019). Management of preeclampsia, severe preeclampsia, and eclampsia at primary care facilities in Bangladesh. Glob Health Sci Pract.

[54] Mpunga Mukendi D, Chenge F, Mapatano MA, Criel B, Wembodinga G. Distribution and quality of emergency obstetric care service delivery in the Democratic Republic of the Congo: it is time to improve regulatory mechanisms. Reprod Health. 2019;16 https://www.ncbi.nlm.nih.gov/pmc/articles/PMC6631736/ Accessed 15 July 2020. 10.1186/s12978-019-0772-z PMC6631736 31307497

[55] Salomon A, Ishaku S, Kirk KR, Warren CE (2019). Detecting and managing hypertensive disorders in pregnancy: a cross-sectional analysis of the quality of antenatal care in Nigeria. BMC Health Serv Res.

[56] Poon LC, Shennan A, Hyett JA, Kapur A, Hadar E, Divakar H (2019). The International Federation of Gynecology and Obstetrics (FIGO) initiative on pre-eclampsia: a pragmatic guide for first-trimester screening and prevention. Int J Gynecol Obstet.

[57] e Holanda Moura SBM, Marques Lopes L, Murthi P, da Silva Costa F. Prevention of preeclampsia. J Pregnancy. 2012;2012 https://www.ncbi.nlm.nih.gov/pmc/articles/PMC3534321/. Accessed 20 Dec 2019. 10.1155/2012/435090 PMC3534321 23316362

[58] Han A, Bujold E, Belizán M, Jaime J, Belizán J, Sharma S (2016). Preventing pre-eclampsia and its complications. The FIGO textbook of pregnancy hypertension.

[59] Althabe F, Chomba E, Tshefu AK, Banda E, Belizán M, Bergel E (2019). A multifaceted intervention to improve syphilis screening and treatment in pregnant women in Kinshasa, Democratic Republic of the Congo and in Lusaka, Zambia: a cluster randomised controlled trial. Lancet Glob Health.

[60] Raney JH, Morgan MC, Christmas A, Sterling M, Spindler H, Ghosh R (2019). Simulation-enhanced nurse mentoring to improve preeclampsia and eclampsia care: an education intervention study in Bihar, India. BMC Pregnancy Childbirth.

[61] Nelissen E, Ersdal H, Mduma E, Evjen-Olsen B, Twisk J, Broerse J, et al. Clinical performance and patient outcome after simulation-based training in prevention and management of postpartum haemorrhage: an educational intervention study in a low-resource setting. BMC Pregnancy Childbirth 2017;17(1):301. doi: 10.1186/s12884-017-1481-7. Accessed 20 Dec 2019. 10.1186/s12884-017-1481-7 PMC5594489 28893211

[62] Casey SE, Mitchell KT, Amisi IM, Haliza MM, Aveledi B, Kalenga P (2009). Use of facility assessment data to improve reproductive health service delivery in the Democratic Republic of the Congo. Confl Health.

